# In Vitro and In Vivo Assessment of PEGylated PEI for Anti-IL-8/CxCL-1 siRNA Delivery to the Lungs

**DOI:** 10.3390/nano10071248

**Published:** 2020-06-27

**Authors:** Alan J. Hibbitts, Joanne M. Ramsey, James Barlow, Ronan MacLoughlin, Sally-Ann Cryan

**Affiliations:** 1 School of Pharmacy & Biomolecular Sciences, Royal College of Surgeons in Ireland, Dublin D02 YN77, Ireland; alanhibbitts@rcsi.ie (A.J.H.); ramseyj@tcd.ie (J.M.R.); RMacLoughlin@aerogen.com (R.M.); 2 Trinity Centre for Biomedical Engineering, Trinity College, Dublin D02 R590, Ireland; 3 Department of Chemistry, Royal College of Surgeons in Ireland, Dublin D02 YN77, Ireland; jambarlow@rcsi.ie; 4 School of Pharmacy and Pharmaceutical Sciences, Trinity College, Dublin D02 PN40, Ireland; 5 Aerogen Ltd. Galway Business Park, Galway H91 HE94, Ireland

**Keywords:** siRNA, vibrating mesh nebuliser, PEI, PEI-PEG, IL-8, inflammation, PEGylation, aerosol, macrophage

## Abstract

Inhalation offers a means of rapid, local delivery of siRNA to treat a range of autoimmune or inflammatory respiratory conditions. This work investigated the potential of a linear 10 kDa Poly(ethylene glycol) (PEG)-modified 25 kDa branched polyethyleneimine (PEI) (PEI-LPEG) to effectively deliver siRNA to airway epithelial cells. Following optimization with anti- glyceraldehyde 3-phosphate dehydrogenase (GAPDH) siRNA, PEI and PEI-LPEG anti-IL8 siRNA nanoparticles were assessed for efficacy using polarised Calu-3 human airway epithelial cells and a twin stage impinger (TSI) in vitro lung model. Studies were then advanced to an in vivo lipopolysaccharide (LPS)-stimulated rodent model of inflammation. In parallel, the suitability of the siRNA-loaded nanoparticles for nebulization using a vibrating mesh nebuliser was assessed. The siRNA nanoparticles were nebulised using an Aerogen^®^ Pro vibrating mesh nebuliser and characterised for aerosol output, droplet size and fine particle fraction. Only PEI anti-IL8 siRNA nanoparticles were capable of significant levels of IL-8 knockdown in vitro in non-nebulised samples. However, on nebulization through a TSI, only PEI-PEG siRNA nanoparticles demonstrated significant decreases in gene and protein expression in polarised Calu-3 cells. In vivo, both anti-CXCL-1 (rat IL-8 homologue) nanoparticles demonstrated a decreased CXCL-1 gene expression in lung tissue, but this was non-significant. However, PEI anti-CXCL-1 siRNA-treated rats were found to have significantly less infiltrating macrophages in their bronchoalveolar lavage (BAL) fluid. Overall, the in vivo gene and protein inhibition findings indicated a result more reminiscent of the in vitro bolus delivery rather than the in vitro nebulization data. This work demonstrates the potential of nebulised PEI-PEG siRNA nanoparticles in modulating pulmonary inflammation and highlights the need to move towards more relevant in vitro and in vivo models for respiratory drug development.

## 1. Introduction

Oligonucleotide therapeutics offer a unique opportunity for accurate and specific disease treatment at a genetic level. To date, these have been investigated in a variety of endogenous and infectious conditions [1,2,3]. Of these, siRNA has gained renewed prominence following the successful clinical approval of Patisiran (ONPATTRO™) by Alnylam Pharmaceuticals for hereditary TTR-mediated amyloidosis (hATTR) [4]. In part, the approval was based on Patisiran’s ability to rapidly and accurately reach its target cells (hepatocytes) by utilising the most appropriate delivery method (i.v. perfusion) [5]. When the i.v. route is not practical or effective, utilising local delivery routes offer a means of vastly increasing the targeting of oligonucleotides and thereby improving the specificity and efficacy [6].

Local delivery is particularly promising as an approach for oligonucleotide therapeutics targeting respiratory diseases. This is especially true for episodes of acute pulmonary inflammation, including acute asthmatic episodes and pathogenic respiratory conditions such as acute respiratory distress syndrome (ARDS) [7,8]. The urgent need to mitigate the effects of respiratory viruses has become especially relevant with the emergence of the COVID-19 pandemic. COVID-19 is clinically manifest in the lungs in ~50% of patients and mortality is strongly linked to cytokine storm-induced ARDS [9,10,11]. In these cases, a fast-acting specific treatment of short duration may be more favourable than a continuous, systemic depression of the innate immune system in an otherwise healthy individual. One potential target for cytokine-induced respiratory distress is the chemotactic pro-inflammatory cytokine IL-8. IL-8 is known to be secreted from pulmonary epithelial cells and is elevated in COVID-19 patients [12,13]. Furthermore, anti-IL-8 therapy is currently under investigation in phase II clinical trials using i.v.-delivered anti-IL-8 monoclonal antibodies in COVID-19 patients [14].

However, the effective delivery of siRNA to the lungs has been hampered by poor delivery device performance. This is especially true in the case of the nebulised delivery of siRNA. Nebulisation offers the benefits of a non-invasive, easy to use device with a large level of flexibility in the dose delivered. It has long been established that air-jet and older ultrasonic nebulisers are unsuited to siRNA delivery due the high cost of the therapeutic cargo and their known deficiencies in output efficiency [15]. Previous tests have indicated that air-jet and ultrasonic nebuliser devices have a considerable discrepancy in their ability to effectively form droplets of the desired size [16]. To address this, there has been a relatively new “3rd generation” of nebulisers developed known as vibrating mesh nebulisers (VMNs) (air-jet and ultrasonic nebulisers being the 1st and 2nd generations, respectively). These nebulisers function in a manner modified from previous ultrasonic nebulisers, whereby a plate containing approximately 1000 tapered holes vibrates at a frequency of roughly 128 kHz, thereby causing the ejection of liquid droplets [17]. Vibrating mesh nebulisers are now becoming widespread and there is investigation into their use for delivery of sensitive therapeutic cargoes, such as proteins [18], miRNA and anti-miRNA target site-blocking oligonucleotides [19,20], mRNA [21] and siRNA [22,23]. Furthermore, the bulk of recent publications indicate that VMNs deliver more drug to the patient with significantly less wastage, than the more prevalent air-jet nebulisers [24,25,26].

In parallel to effective device-borne delivery to the lungs, the successful development of a locally delivered siRNA therapy for pulmonary conditions must contend with the challenges of the lung micro-environment. Major barriers to local pulmonary delivery include the mucociliary clearance action of the ciliated epithelial cells and the presence of mucus and alveolar fluid in different parts of the airways [27,28]. Particles that are deposited on the ciliated cells are rapidly removed by muco-ciliary clearance and are eventually coughed up or swallowed. This mucus lines the respiratory epithelium from the nasal cavity to the terminal bronchioles [29]. Efforts to overcome the mucus barrier and improve the efficacy of delivered drugs has led to the development of “mucus penetrating particles”. Previous approaches to modifying cationic polymers for siRNA delivery have drawn inspiration from naturally occurring molecules such as viral proteins [30]. Of particular interest is the use of poly(ethylene glycol) (PEG)-modified nanoparticles, which has demonstrated much potential in this regard [31]. Recent research within our group has focused on developing nanoparticle carriers to better deliver siRNA to the pulmonary epithelium [23,32,33]. Specifically, we have previously developed a block co-polymer of 25 kDa branched polyethyleneimine (PEI) conjugated to a high degree (80%) with 10 kDa linear poly(ethylene glycol) (PEG). This PEI-LPEG was capable of higher levels of gene knockdown in fully polarised Calu-3 epithelial monolayers compared to the PEI controls, with limited cellular toxicity evident [32]. In this study, this PEI-LPEG polymer will be further investigated for its potential to deliver anti-IL-8 siRNA to the lungs via a vibrating mesh nebuliser. Given its prominent role in modulating pulmonary inflammation, the successful nebulisation of anti-IL-8 siRNA would serve as a promising proof of concept in combatting pulmonary inflammation at the genetic level.

To achieve this, PEI-LPEG siRNA and PEI siRNA nanoparticles were nebulised using a vibrating mesh nebuliser, and their post-nebulisation stability was assessed in terms of their physicochemical properties and their ability to facilitate gene knockdown. Cell studies first focused on validating the sequence specificity of anti-IL-8 siRNA in non-nebulised transfections. Following this, the post-nebulisation efficacy was investigated following passage through a twin-stage impinger (TSI). This was first validated at a genetic level using the glyceraldehyde 3-phosphate dehydrogenase (GAPDH) house-keeping gene before assessing the changes in IL-8 protein levels. Finally, anti-CXCL-1 (rat IL-8 homologue [34]) siRNA nanoparticles were assessed in an in vivo pilot study using a rat model of acute pulmonary inflammation involving intra-tracheal intubation, siRNA delivery and subsequent lipopolysaccharide (LPS) challenge.

## 2. Materials and Methods

### 2.1. Materials

All the cell culture reagents were obtained from Invitrogen Corporation/ThermoFisher Scientific (CA, USA) unless otherwise stated. The Calu-3 bronchial epithelial cell line was obtained from the American Tissue Type Culture Collection (ATCC) and used at passages 20–40. All the poly (ethylene glycol) molecules were obtained from Iris Biotech (Marktredwitz, Germany). The siRNA sequences for human GAPDH, β-actin and IL-8 were obtained from Qiagen UK and were diluted 1:10 in TE buffer as directed. The specific sequences remained the proprietary knowledge of Qiagen. The siRNA sequences for rat CXCL-1 (5′ UAACGAGAUAUUUAACGCCCCC 3′) were obtained from (Riboxx GmbH, Radebeul, Germany), and the siGENOME non-targeting siRNA #2 (5′ UAAGGCUAUGAAGAGAUAC 3′) scrambled sequence controls were obtained from Dharmacon (now Horizon Discovery, Cambridge, UK). All the other general chemicals and reagents used were of the highest grade possible and were obtained from Sigma-Aldrich Company Ltd. (Wicklow, Ireland) unless otherwise stated.

### 2.2. PEI and PEI-PEG siRNA Nanoparticle Formation

Branched 25 kDa PEI was modified with 10 kDa linear PEG by the reaction of succinimidyl-activated PEG (PEG-SSA) with PEI under slightly basic aqueous conditions as previously described [32]. Briefly, 1 g of 25 kDa PEI was dissolved in 25 mL of phosphate buffered saline (pH 8). Following this, 500 mg of 10 kDa linear PEG-SSA was dissolved in 5 mL of 99.9% dimethyl sulfoxide (DMSO). This was added dropwise with stirring to the PEI solution. The reaction proceeded for 4 h at room temperature before being stopped by the addition of 60 mL of deionised water. The reaction mixture was transferred to Cellu•Sep H1 membranes (25 kDa MWCO) (Orange Scientific, Braine-l’Alleud, Belgium) and dialysed overnight in excess deionised water to remove unreacted components before being lyophilised. The final products were then analysed for form and purity using ^1^H and ^13^C NMR spectroscopy (Bruker Avance 400) and gel permeation chromatography (GPC) (Perkin Elmer, Dublin, Ireland) with electronic light scattering detection (Agilent Technologies, Cork, Ireland).

PEI and PEI-PEG Polymer-siRNA complexes were formed using PEI nitrogen to RNA phosphate (N/P) in ratios of 15 and 7. Briefly, the appropriate amount of polymer was added to an siRNA solution (20 μM) to yield a final concentration of 2 μM siRNA, vortexed for 10 s and incubated for polyplex formation for 30 min. Following this, the solutions were diluted using PBS.

### 2.3. Laser Diffraction and Surface Tension Analysis of siRNA Nanoparticles nebulised Using Vibrating Mesh Nebulisers

The siRNA nanoparticles were nebulised and the droplet size distributions, described by a volumetric median diameter (VMD), were measured by a Malvern Spraytec particle size analyser for the droplets produced (Malvern Instruments Ltd., Malvern, Worcestershire, UK) with the RT Sizer software (version 5.60).

A 5 L/min vacuum flow was implemented through the system, ensuring a laminar flow and reducing the artificial droplet size growth through collision with other droplets. The vacuum also ensured that the droplets passed through the laser beam only once. The centre of the emitted aerosol plume was directed through the centre of the laser beam to increase the accuracy of data acquisition.

Data acquisition was performed as previously described [23], beginning when beam obscuration exceeded 3% and continued until the end of dosing. The data acquisition rate was set to 500 Hz, which is 500 individual readings per second taken to characterise the droplet size distribution. The data reported for each individual measurement is an average of the individual readings recorded over the course of the dose. In order to verify the accuracy of the generated data, the Spraytec analyser’s laser diffraction apparatus was tested with a reference reticle (Malvern Instruments Ltd., Malvern, Worcestershire, UK). The droplet size is described by volumetric median diameter (Dv_50_) and the fine particle fraction (FPF) (percentage of droplets less than 5 μm in size).

The surface tension of the siRNA nanoparticle solutions was also examined using a ring/plate tensiometer (LAUDA Scientific GmbH, Lauda-Königshofen, Germany) at room temperature according to the manufacturer’s instructions. In all cases, the changes in output rates and fluid surface tension were compared to PBS controls.

### 2.4. Post-Nebulisation siRNA Nanoparticle Size Distribution

The PEI and PEI-PEG siRNA nanoparticles were formed as previously described using 7.5 μg of siRNA and diluting with PBS to a final volume of 5 mL Polyplex. The siRNA samples were nebulised into a glass impinger at 60 L/min using an Aerogen^®^ Pro vibrating mesh nebuliser (Aerogen, Galway, Ireland). Before nebulisation, PBS was added to the upper (Stage A, 3 mL) and lower (Stage B, 7 mL) stages. Washes were quantitatively collected using PBS with the Device, Throat, Stage A and Stage B made to 5, 5, 10 and 25 mL respectively.

A size analysis of the siRNA polyplexes was performed using a Malvern Nano-ZS Zetasizer. Pre-nebulisation samples underwent a 1 in 10 dilution in dH_2_O before addition of PBS. Post-nebulisation, rinsed samples were concentrated by centrifuging out excess PBS using Centrisart 1 Centrifugal UF Units (20 kDa MWCO, Sartorius). Despite this, due to the high level of dilution that occurred and the strong ionic character of PBS, it was not possible to accurately measure the zeta potentials of the post nebulisation samples. Size analysis programmes consisted of five separate scans which contained a minimum of 15 sub-scans.

### 2.5. Non-Nebulised Anti-IL-8 siRNA Transfection and Protein Knockdown in Polarised Calu-3 Cells

Calu-3 cells were seeded in 12 mm transwell chambers at 5 × 10^5^ cells/well cultured in complete media of 50:50 DMEM:Ham’s F-12 (10% FBS and 1% pen/strep) in at a liquid-liquid interface for 48 h and for a further 10–12 days at an air/liquid interface. A trans-epithelial electrical resistance (TEER) value of >500 Ωcm^2^ was taken as a sign of tight junction formation.

On the day of transfection, 0.6 µg/well (360 nM) of siRNA nanoparticles in PBS were formed as previously described. The siRNA nanoparticles were administered directly to each well and incubated for 24 h at 37 °C and 5% CO_2_. Following this, the apical (600 µL) and baso-lateral (1.2 mL) layers of the transwells were aspirated off and collected for IL-8 ELISA analysis (Invitrogen via Bio-Sciences, Dublin, Ireland) according to manufacturer’s instructions and later normalised to account for differences in dilution. Sequence specificity was demonstrated by use of non-complexed PEI or PEI-LPEG and GAPDH-siRNA negative controls.

In addition to this, the TEER value fluctuations over the 24 h of transfection were recorded using an EVOM voltohmmeter (World Precision Instruments, Stevenage, UK) at various time points for analysis. Briefly, a baseline reading was established prior to transfection by equilibrating the apical layer in PBS for 45 min at 37 °C prior to reading. Following the addition of the siRNA nanoparticle PBS, readings were taken at 30 min and at 1, 2, 4 and 24 h and compared to the PBS-only treated controls.

### 2.6. Nebulised siRNA Nanoparticle Delivery onto Polarised Calu-3 Cells

Calu-3 cells were seeded in 6.5 mm transwell plates at a density of 2.5 × 10^5^ cells/well using the previously described methods for Calu-3 culture in transwell plates at liquid-liquid and air-liquid interfaces. Cell monolayers were used for the transfection studies once a TEER of >500 Ωcm^2^ was established [35].

**Figure 1:**
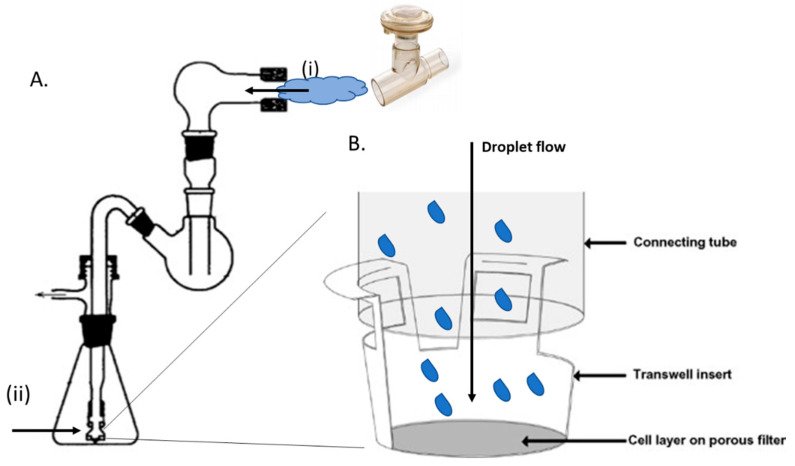
Schematic overview of (**A**) twin-stage impinger set up with nebulised siRNA nanoparticles entering at position (i) and impacting on the cell monolayer at (ii). (**B**) Enhanced view of nebuliser droplet expulsion onto Calu-3 cells on a transwell insert with the airflow redirected laterally through the openings of the transwell walls (adapted from [35], with permission from Elsevier, 2020).

A glass TSI was assembled as previously described by Grainger et al. [35], apart from the absence of solution in the lower chamber. An Aerogen^®^ Pro vibrating mesh nebuliser was sealed into place with parafilm at the entrance of the TSI (Figure 1A(i)). The adapter piece (Figure 1A(ii)) was removed and parafilm was wrapped around the base of the connecting tube to produce an attachment surface for the transwell insert. The transwell insert was then pushed onto the connecting tube until the tapered internal walls of the insert fastened firmly onto the parafilm (Figure 1B). Thus, air flowing down the connecting tube was diverted through the lateral ports of the insert. This caused particles to exit the air stream through inertial impaction and deposit onto the cell layer. Following this, preformed PEI/PEI-LPEG siRNA nanoparticles at a concentration of 360 nM were delivered using either 0.6, 1.2 or 2.4 μg of siRNA per well. The vacuum pump was run at 60 L/min and allowed to run for 30 s past complete sample nebulisation. The nebuliser was rinsed by nebulising 3 times with 500 μL of PBS between doses with no transwell attached to the lower section. Furthermore, the TSI itself was disassembled and rinsed out with sterile deionised water when switching between the PEI and PEI-LPEG samples. Following the nebulisation of all samples, the cells were incubated for 24 h at 37 °C and 5% CO_2_. GAPDH siRNA was first delivered to validate the system using gene expression prior to the delivery of the anti-IL-8 siRNA and the protein expression analysis.

Following 24 h of incubation, the basal secretions were collected and the apical layer of cells was washed and collected with an equivalent amount of sterile PBS. The GAPDH gene knockdown was then assessed using real-time RT-PCR. RNA was extracted using the RNeasy Micro Kit (Qiagen, UK) following the manufacturer’s instructions. cDNA for the real-time PCR was synthesised from RNA using the high capacity reverse transcription kit (Applied Bio-systems via Bio-Sciences, Dublin, Ireland) according to the manufacturer’s instructions. A quantitative PCR was performed in 200 μL tubes using a Rotor-gene 6000 (Corbett Research, UK, now Qiagen, Manchester, UK) thermal cycler with the real-time detection of fluorescence. The PCR was conducted in a volume of 25 μL using Rotor-Gene SYBR Green RT-PCR kit (Qiagen, Manchester, UK). The percentage knockdown of GAPDH was then quantified using the Comparative Quantitation software supplied by Corbett Research for the Rotorgene. In all the experiments, human β-actin was used as a housekeeping gene to normalise the expression. In the case of anti-IL-8 siRNA delivery, the protein levels were assessed as previously described using an IL-8 ELISA.

### 2.7. CXCL-1 Knockdown in an LPS-Stimulated Rat Model Using Intra-Tracheally Delivered siRNA Nanoparticles

#### 2.7.1. Endotracheal Intubation

Following institutional and governmental ethical approval (RCSI ethical approval: REC743, animal licence ref: B100/4331), female Sprague Dawley rats (200–225 g) received an intraperitoneal (I.P.) administration of anaesthetic (xylazine 10 mg/kg and ketamine 85 mg/kg) and were returned to their home cage until the anaesthetic took effect. A tail and toe pinch were used to assess the depth of anaesthesia. The anaesthetised rats were positioned supine on a BioLite rodent intubation stand (Kent Scientific, Torrington, CT, USA) suspended from the front incisors. The tongue was extended and moved to one side using forceps. A small animal laryngoscope was used to illuminate the vocal cords, epiglottis and opening to the trachea as well as to hold the tongue in place. Once the airway of the rat had been visualised, a Penn-Century IA-1C MicroSprayer^®^ (Penn-Century Inc, Wyndmoor, PA, USA) with a flexible stainless steel delivery tube of 0.64 mm (23-gauge) attached was used to access the lungs for siRNA nanoparticle or LPS delivery.

#### 2.7.2. Intratracheal Administration of siRNA Nanoparticles

Prior to endotracheal intubation, PBS, LPS or 75 μg of siRNA nanoparticles (200 μL) were loaded into the MicroSprayer. This was achieved by aspirating the entire pre-prepared 200 μL volume into the MicroSprayer and attaching 4 spacer clips corresponding to 50 μL each attached to the handle of the MicroSprayer. The dead volume was subsequently expelled by pressing down on the MicroSprayer handle until only 200 μL remained. Once the rat had been successfully intubated, approximately 200 μL of the sample was delivered by pressing down the handle of the MicroSprayer quickly and steadily to completion and holding for approximately 10 s to ensure full release of the sample. The MicroSprayer was immediately removed and the rats were administered 15 μL of 5 mg/mL atipamezole hydrochloride (Sedastop™) diluted to 150 μL using sterile PBS via I.P. injection to aid recovery and were placed on their sides in a heated incubator until consciousness was regained. The rats were then moved to a room with constant temperature set at 37 °C for 21 h with access to food and water ad libitum. Then, 21 h after the initial treatment, the rats underwent the intratracheal administration of either 200 μL of sterile PBS or 1 mg/mL of LPS (from *E. coli* 055:B5) by the same procedure. The animals were not administered Sedastop™ on this occasion and were placed in the incubator for 3 h. After 3 h, the rats were sedated again if necessary and then sacrificed via cervical dislocation for tissue harvesting.

#### 2.7.3. In Vivo Tissue Harvesting

Once the rats were euthanised, the thoracic cavity was opened. The lungs and heart were excised en bloc. One bronchus was clamped to allow BAL collection from one side of the lungs only. 1 mL of saline was added to one side of the lungs through the trachea using a 20-gauge catheter with the needle removed and a 5 mL syringe. Saline was collected and a further 1 mL of fresh saline was added. This procedure was repeated until the lung had been washed with 5 mL of saline. The remaining lobes were collected for RT-PCR in 5 mL of RNAlater™ (ThermoFisher via Bio-Sciences, Dublin, Ireland).

#### 2.7.4. Bronchoalveolar Lavage (BAL) and Differential Cell Count

The collected BAL fluid was centrifuged at 1× *g* for 10 min at 4 °C. Supernatants were collected as BAL fluid and stored at −80 °C. The cell pellets were resuspended in 1 mL of red cell lysis buffer and centrifuged at 1× *g* for 10 min at 4 °C. The supernatants were discarded and the cell pellets were resuspended in 100 μL of saline. An amount of 10 μL of cell suspension was mixed with 90 μL of trypan blue and the total cell counts taken using a haemocytometer. The remainder of the cell suspension was spun for 5 min at 500 rpm onto Shandon coated microscope slides (Fisher Scientific, Dublin, Ireland) using Shandon filter cards and Shandon Cytospin^®^ 2. The cells were fixed and stained using Speedy-Diff 203 cell stain kit (Clin-Tech Ltd., Guildford, UK). Briefly, the slides were immersed for a few seconds in speedy-diff fixative (coloured methanol) five times then immersed for a few seconds in Speedy-Diff A (buffered Eosin Y) at least 5 times followed by blotting and washing in PBS and finally immersed in Speedy-Diff B (buffered azur/methylene blue) at least 5 times, blotted and washed. The slides were dried and the differential cell counts were obtained using a CETI light microscope (Medline Scientific, Chalgrove Oxon, UK) at 100× magnification under oil immersion. The macrophages and neutrophils were counted in 5 random fields and the total differential cell counts were calculated.

#### 2.7.5. In Vivo Cytokine Expression

A Rat Demonstration 7-Plex Ultra-Sensitive Kit assay (MesoScale Discovery, Rockville, MD, USA was used to simultaneously detect IFN-γ, IL- 1β, IL-4, IL-5, IL-13, KC/GRO/CXCL-1 and TFN-α cytokines. The assay was set up and run according to the manufacturer’s instructions. Briefly, 25 μL of provided Diluent 6 was added to each well and the plate was sealed and incubated at room temperature with vigorous shaking (300–1000 rpm) for 30 min. An 8-point serial dilution of Rat Demonstration 7-Plex calibrator blend (40000-0 pg/mL) was prepared in 1% BSA in PBS (for BAL fluid calibration). The BAL fluid samples were mixed with 1% BSA to reduce protein adherence to the micro-tubes. Standards and BAL fluid (25 μL) were added in duplicate to wells and the plate was sealed and incubated for 2 h at room temperature with vigorous shaking. The plate was washed 3 times with PBS-T followed by the addition of 25 μL of 1× detection antibody solution. The plate was resealed and vigorously shaken at room temperature for 2 h. The washing 3 times in PBS-T was repeated. An amount of 150 μL of 2× Read Buffer T (4× Read Buffer T diluted in dH_2_O) was added to each well and the plate was immediately analysed on a SECTOR Imager. Standard curves were produced and the concentrations of analytes for each sample determined using the MSD DISCOVERY WORKBENCH^®^ software.

#### 2.7.6. Histopathology

The fixed lung tissue was embedded in paraffin and the sections were mounted onto slides. The paraffin was removed by immersing slides in HistoChoice^®^ Clearing Agent for at least 30 min. The tissue sections were hydrated by immersing them for a few seconds in 100% ethanol followed by 90%, 70% and 50% ethanol and finally dH_2_O. After hydration, the sections were stained with haematoxylin for 15 min followed by rinsing in dH_2_O. The sections were differentiated in 1% acid alcohol to remove excess haematoxylin by immersing for a few seconds in 50% ethanol, then 70% ethanol followed by acid alcohol (1% concentrated hydrochloric acid in 70% ethanol (v/v)). The sections were then immersed in 70% ethanol followed by rinsing in dH_2_O. The sections were immersed in alkaline ammonia water (0.2% potassium bicarbonate and 2% magnesium sulphate) and rinsed by running under tap water for 10 min. Following rinsing, the sections were stained with eosin (5 min) and then immersed for a few seconds in dH_2_O. The sections were then dehydrated by immersing for a few seconds in 50%, then 70%, 90% and finally 100% ethanol followed by a short immersion in HistoChoice^®^ Clearing Agent. Coverslips were mounted onto the slides with DPX mounting medium. The lung sections were examined for inflammation by light microscopy. The degree of neutrophil-rich inflammation was scored as − (absent), +/− (very mild), + (mild) or ++ (moderate). Scores were then assigned per treatment group. Photomicrographs were acquired for each “score” using a Nikon Eclipse E600 microscope at 40×, 200× and 600×.

### 2.8. Statistical Analysis

All the samples were run in triplicate and the experiment was repeated on 3 independent occasions unless otherwise stated. Statistical significance was determined for in vitro assays using the GraphPad Prism 5 software and the one or two-way ANOVA method of statistical analysis using Bonferroni multiple comparison tests analysis in all cases. For the in vivo work, significance was calculated using the Kruskal–Wallis test and Dunn’s post-hoc test. The data was expressed ± the standard deviation at all times (SD). The results were deemed to be statistically significant (*) where the *p* values were found to be <0.05, very significant (**) at *p* < 0.01 and extremely significant (***) at *p* < 0.001.

## 3. Results

### 3.1. PEI-LPEG Characterisation

Following purification, the PEI-LPEG was analysed via GPC, where it was found that the final product demonstrated a faster elution time than either the PEI or LPEG starting materials (Figure S1). This indicated a larger molecular weight co-polymer had been effectively synthesised. Confirmation of the successful conjugation was also carried out using ^13^C NMR, which highlighted the presence of the carboxylic bonding between the PEI and LPEG at 164 ppm (Figure S2). Finally, the % grafting was estimated using ^1^H NMR and the integration of the PEI present at 2.5 ppm and the PEG present at 3.5 ppm (Figure S3). This was approximately 80% grafted, as was previously the case when the initial in vitro work was reported [32].

### 3.2. Effect of VMN Nebulisation on PEI and PEI-PEG siRNA Nanoparticle Size

In order to establish an effective inhaled siRNA therapy, it is important to examine the effect of nebulisation on the siRNA nanoparticle size and to establish that, on reaching their site of action, the siRNA nanoparticles were of the desired size for cell endocytosis.

**Figure 2:**
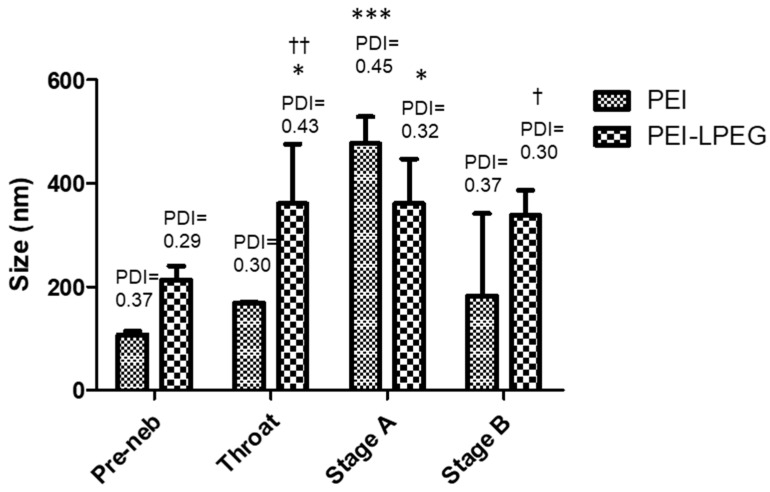
Size distribution and polydispersity index (PDI) of the nebulised polyethyleneimine (PEI) and Poly(ethylene glycol) (PEG)-modified 25 kDa branched polyethyleneimine (PEI-LPEG) siRNA nanoparticles at N/P = 15 following nebulisation and collection in each section of a twin-stage impinger (TSI). (* significance vs. pre-neb samples, † significance PEI vs. PEI-LPEG, 2-way ANOVA, *n* = 5 ± SD, † * *p* < 0.05, †† *p* < 0.01 *** *p* < 0.001).

The siRNA nanoparticles underwent particle sizing before nebulisation. Following nebulisation using a vibrating mesh nebuliser (VMN) and passage through the TSI, the siRNA nanoparticles were collected from the throat and Stage A and B of the TSI and resized. It was found that the vibrating mesh nebulisation of siRNA nanoparticles did not result in significant changes to the nanoparticle size in the respirable fraction (Figure 2).

In the case of the PEI siRNA nanoparticles, the greatest increases in nanoparticle size and polydispersity were found in stage A, which corresponds to the non-respirable fraction. However, the nanoparticle size was found to be unchanged in samples collected from stage B of the TSI, corresponding to the respirable fraction.

Similarly, the PEI-LPEG siRNA nanoparticles collected in the throat and upper chamber were found to have significant increases in size and polydispersity compared to the un-nebulised samples. However, the nanoparticles found in stage B demonstrated no significant changes. On comparing the PEI and PEI-LPEG siRNA nanoparticles, the PEGylated particles were significantly larger than the unmodified PEI in the lower stage (Stage B) of the TSI. However, the polydispersity indices for both did not demonstrate any significant differences.

### 3.3. siRNA Nanoparticle Droplet Size Analysis

The Aerogen Pro vibrating mesh nebuliser was used to nebulise the PEI and PEI-LPEG siRNA nanoparticles, and the VMD and %FPF was determined and compared to the nebulisation of PBS using the same device (Table 1). On the examination of the VMD and %FPF of each siRNA nanoparticle suspension, it was found that there were no significant changes when compared to the PBS controls. The VMD of the droplets containing siRNA nanoparticles remained around ~5 µm in all cases, which is within the required size range for successful delivery to the deep lungs [36]. In addition, the %FPF of each nebulised siRNA nanoparticle sample remained between 55% and 60%. However, on examining the changes in output rate, the PEI-LPEG siRNA N/P = 15 nanoparticle samples demonstrated a 33% drop in output efficiency compared to the PBS controls. The surface tension of the PEI and PEI-LPEG siRNA nanoparticle samples at the concentrations tested was measured, as this has been found to impact on the nebulisation of liquids. The PEI-LPEG-siRNA nanoparticle system had a decreased surface tension, although this was found not to be statistically significant compared to the other groups.

### 3.4. Anti-IL-8 siRNA Transfection of Calu-3 Monolayers

To examine the suitability of PEI/PEI-PEG siRNA nanoparticles for the delivery of a potentially therapeutic anti-inflammatory sequence, anti-IL-8 siRNA was directly delivered to fully polarised Calu-3 monolayers (N/P = 15). For a more meaningful assessment of the potential therapeutic effect, the IL-8 expression in Calu-3 cells was investigated at the protein level using an IL-8-specific ELISA. This also allowed for the examination of protein expression in both the apical and basal secretions of the Calu-3 cells grown on Transwell™ inserts.

**Figure 3:**
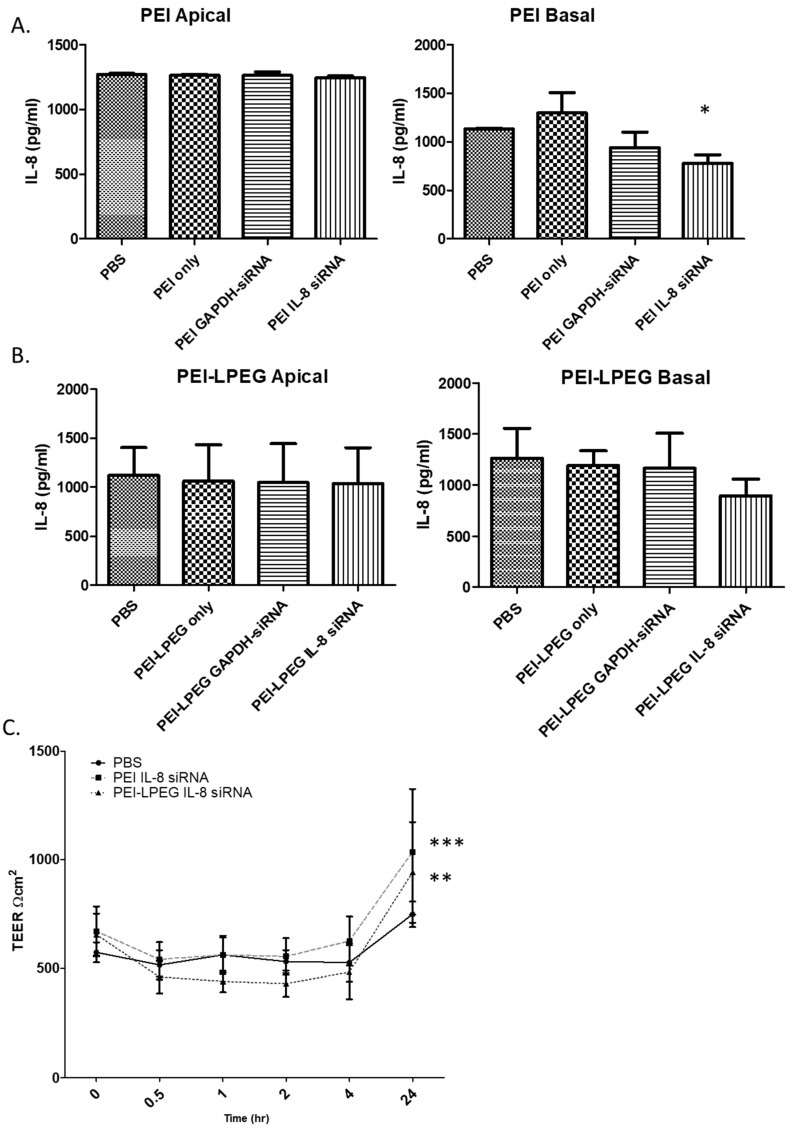
ELISA analysis of the IL-8 knockdown efficiency of (**A**) PEI and (**B**) PEI-LPEG siRNA nanoparticles following direct administration onto fully polarised Calu-3 monolayers at N/P = 15. (**C**) Analysis of trans-epithelial electrical resistance (TEER) fluctuations in PEI and PEI-LPEG siRNA nanoparticle (N/P = 15) over 24 h (significance vs. PBS-treated samples, 1 and 2-way ANOVA, *n* = 3 ± SD, * *p* < 0.05, ** *p* < 0.01, *** *p* < 0.001).

On the analysis of the PEI anti-IL8 siRNA nanoparticle-treated samples, it was found that there were no significant changes in IL-8 secretion in any of the apical samples. However, there were significant changes in the IL-8 secretion in the basal samples (Figure 3A) detected for PEI anti-IL-8 siRNA nanoparticles (780 pg/mL ± 85.08 pg/mL) in comparison to the PBS samples (1131 pg/mL ± 8.95 pg/mL). The administration of the anti-GAPDH siRNA nanoparticles or an equivalent concentration of free PEI (as controls) did not result in any significant changes in the IL-8 secretion.

On the analysis of the PEI-LPEG siRNA nanoparticle-treated cells, decreases in basal secretions of IL-8 were also observed (Figure 3B). However, these were not found to be statistically significant. Similar to the PEI data, the administration of PEI-LPEG anti-GAPDH siRNA control nanoparticles did not cause any significant changes in the IL-8 expression. Finally, the administration of PEI-LPEG in solution at concentrations equivalent to N/P = 15 nanoparticles to Calu-3 cells did not result in a significant increase in IL-8 secretion, as was also seen with the equivalent PEI treated samples.

As a means of investigating the effect that the administered siRNA nanoparticles had on the Calu-3 cell monolayer tight junctions, the TEER values of the treated cells were taken at regular intervals and compared against PBS-treated cells (Figure 3C). Compared to the PBS-treated samples, the administration of PEI and PEI-LPEG siRNA nanoparticle solutions did not significantly alter the TEER integrity for the first 4 h post-treatment. However, a comparative treatment with PEI-LPEG siRNA nanoparticles resulted in a greater, but non-significant, decrease (26% decrease at 4 h) in the TEER values compared to the PBS (8% decrease at 4 h) or PEI siRNA nanoparticle (7% decrease at 4 h)-treated samples. Interestingly, the greatest drop in TEER in the PEI siRNA nanoparticle-treated samples occurred at 30 min post-treatment, whereas this point was not reached until 2 h post-treatment in the PEI-LPEG siRNA nanoparticle-treated groups. Finally, the TEER values of both the PEI and PEI-LPEG nanoparticles were also found to recover and were significantly higher than the PBS controls at 24 h post treatment. This represents a recovery of tight junction integrity in all the samples and an ability to withstand the stresses involved in siRNA nanoparticle delivery.

### 3.5. Nebulisation of siRNA Nanoparticles onto Polarised Calu-3 Cultures Using a Twin Stage Impinger In Vitro Lung Model

**Figure 4:**
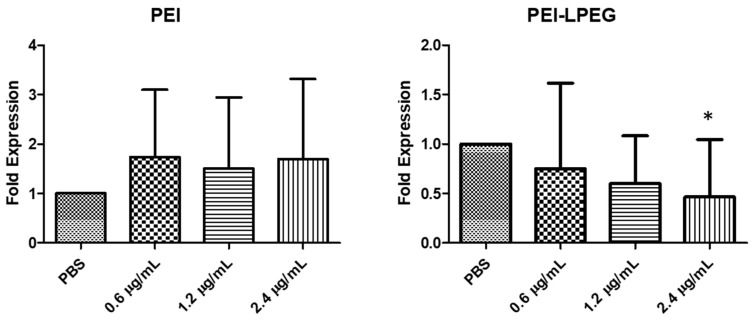
GAPDH genetic knockdown following nebulisation of PEI and PEI-LPEG siRNA nanoparticlesthrough a twin-stage impinger over a range of siRNA doses. At 2.4 µg of siRNA/well, PEI-LPEG demonstrated up to 55% gene inhibition (±15%) and the expression was significantly lower than the PBS-treated controls (significance vs. PBS, 1 way ANOVA, *n* = 5 ± SD, * *p* < 0.05).

In order to comprehensively validate the TSI system, PEI and PEI-LPEG siRNA nanoparticles at N/P = 15 containing 0.6, 1.2 or 2.4 μg/well of anti-GAPDH siRNA were nebulised in five independent experiments. For comparison, 0.6μg/well of siRNA was the standard amount used in previous non-nebulised transfections in Section 3.3. Following the real-time RT-PCR analysis of GAPDH inhibition, there was a significant difference in transfection efficiencies between the PEI and PEI-LPEG-treated samples (Figure 4). The PEI siRNA nanoparticle-treated samples displayed highly variable levels of GAPDH knockdown. Even at the highest doses, the nebulised PEI-mediated GAPDH expression was 1.69-fold (±1.62) that of the PBS-treated cells. In contrast, the nebulised PEI-LPEG siRNA nanoparticles demonstrated significantly greater levels of GAPDH knockdown versus the PBS-treated controls at higher doses. In 2.4 μg/well siRNA nanoparticle-treated samples, the GAPDH expression was found to be 0.46 (±0.58) that of PBS-treated cells. This was significantly lower than both the PBS controls and the corresponding PEI siRNA nanoparticle-treated sample (* *p* < 0.05) when analysed via a 2-way ANOVA.

### 3.6. Nebulisation of Anti-IL-8 siRNA Nanoparticles Using a Twin Stage Impinger In Vitro Lung Model

**Figure 5:**
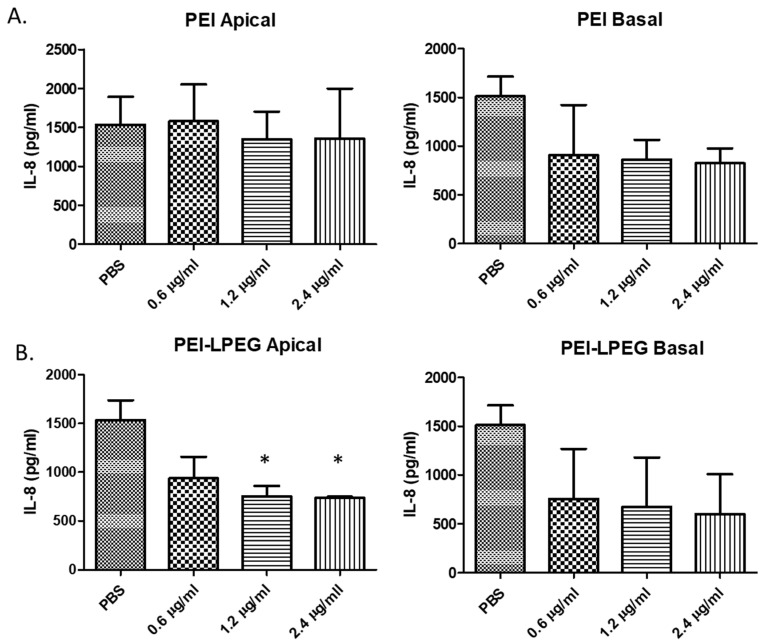
ELISA analysis of the IL-8 knockdown efficiency of (**A**) PEI and (**B**) PEI-LPEG siRNA nanoparticles following nebulisation through a TSI onto fully polarised Calu-3 monolayers at N/P = 15 (significance vs. PBS-treated samples, 1 way ANOVA, *n* = 3 ± SD, * *p* < 0.05).

Following the initial validation of the anti-IL-8 siRNA sequence specificity in standard transfection experiments and the validation of the TSI set-up using anti-GAPDH siRNA, the IL-8 siRNA nanoparticles were administered via the TSI (Figure 5). In the case of PEI anti-IL-8 siRNA nanoparticle-treated cells, protein inhibition was again observed in the basal secretions (Figure 5A). Using 1.2 and 2.4 µg siRNA doses, the IL-8 expression was reduced by up to 45% compared to the PBS-treated controls (824 pg/mL ± 152 SD vs. 1513 pg/mL ± 201 SD, respectively) but were not found to be statistically significant.

In contrast, the nebulised doses of PEI-LPEG siRNA demonstrated IL-8 reduction in both the apical and basal samples (Figure 5B). Significant decreases in IL-8 secretion of up to 50% were seen in apical secretions as well as decreases of up to 40% in basal secretions (non-significant). This demonstrated that only the nebulised PEI-LPEG anti-IL-8 siRNA particles were capable of eliciting a significant inhibitory effect at the protein level.

### 3.7. In Vivo Assessment of siRNA Nanoparticle Knockdown of CXCL-1 Expression in a LPS-Stimulated Rat Model

The intra-tracheal instillation of siRNA nanoparticles in a LPS-stimulated rat model of acute inflammation was investigated in a pilot study. Based on the promising data obtained from in vitro monolayer culture experiments, N/P = 15 was chosen as the N/P ratio, with a siRNA dose of 75 µg extrapolated from a previous in-house study [37]. However, the N/P ratio was reduced due to practical considerations and N/P = 7 was chosen, as it was more comparable with the prior literature [38,39,40]. The animals were treated with PBS-PBS (*n* = 3), PBS-LPS (*n* = 4), PEI-non-targeting (NT) siRNA (*n* = 3), PEI-anti-CXCL-1 siRNA (*n* = 4), PEI-LPEG NT-siRNA (*n* = 3) or PEI-LPEG anti-CXCL-1 siRNA (*n* = 4).

#### 3.7.1. CXCL-1 Gene and Protein Expression

**Figure 6:**
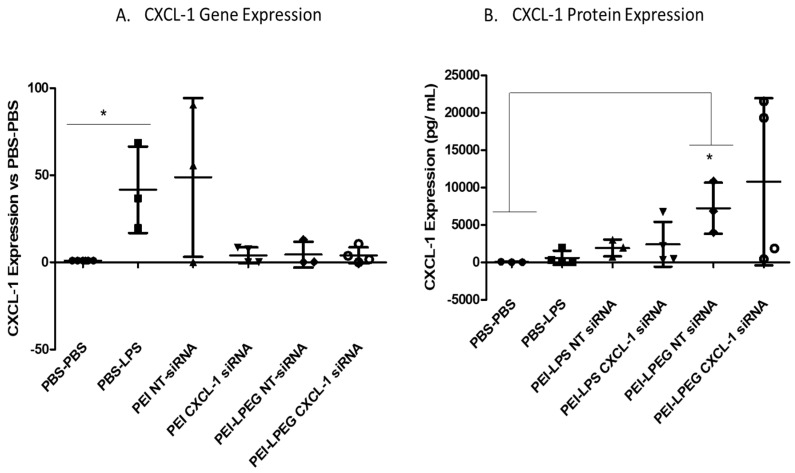
CXCL-1 (**A**) gene and (**B**) protein expression in lipopolysaccharide (LPS)-stimulated rat lungs. Animals were intra-tracheally administered 75 µg of non-targeting (NT) siRNA or anti-CXCL-1 siRNA (N/P = 7) 21 h prior to stimulating with 200 µg of LPS. Animals were euthanised 3 h post-LPS stimulation. CXCL-1 expression was normalised to the β-actin control gene expression (Kruskal–Wallis test and Dunn’s post-hoc test, * *p* < 0.05, min of *n* = 3 ± SD).

Following the LPS administration, there was a significant increase in the CXCL-1 gene expression observed at 3 h post-stimulation (41-fold ± 24.81) compared to the PBS-treated controls (Figure 6A). PEI NT siRNA nanoparticles demonstrated a similar increase in the CXCL-1 gene expression of 48-fold (±45.60). On examination of the PEI anti-CXCL-1 siRNA nanoparticle-treated rats, there were large decreases in the CXCL-1 gene expression evident compared to both the PBS-LPS and NT-siRNA controls. In both cases, there was a 10-fold decrease in the average gene expression between the PEI anti-CXCL-1-treated animals and the control-treated animals (40- vs. 4-fold increases in CXCL-1 expression). However, while the delivery of anti-CXCL-1 siRNA had effectively reduced the gene expression to a point of insignificance when compared to the negative controls, it was not significantly less than the expression in PEI NT siRNA-treated samples due to the variability in responses in this group.

In the case of the PEI-LPEG siRNA nanoparticle-treated groups, both the non-targeting (NT) and anti-CXCL-1 siRNA-treated groups demonstrated 10-fold decreases in the CXCL-1 gene expression compared to the PBS-LPS samples (4- vs. 40-fold respectively). While the gene expression was significantly decreased versus the LPS-treated controls, there were no significant differences between the targeted and non-targeted siRNA-treated animals. This indicated that the effect was not solely related to siRNA-mediated protein inhibition. This was unexpected, considering the PEI-LPEG polymer has been previously validated in vitro as causing no off-target effects in previous work [32] as well as through the results in this study.

The CXCL-1 protein expression in the BAL fluid of treated rats was then examined as part of the 7-plex multi-analyte array (Figure 6B). The results found that, at the early timepoint of 3 h post LPS challenge, only the PEI-LPEG NT siRNA-treated groups demonstrated significantly higher levels of CXCL-1 secretion when compared to the PBS-PBS samples. However, there were no significant differences compared to the PBS-LPS-treated control animals.

The results for each remaining cytokine of the multi-plex array were analysed and the test samples were compared for cytokine expression against the PBS-LPS-treated control animals (Figure S4). Overall, it was found that the administration of siRNA nanoparticles did not lead to statistically relevant changes in cytokine levels compared to the PBS-LPS-treated animals. When compared with the PBS-PBS-treated animals, there were similarly few significant changes in cytokine expression. The PEI-LPEG NT siRNA-treated animals were found to have a significantly higher IL-5 expression compared to the PBS-PBS control. The PEI-LPEG NT and anti-CXCL-1 siRNA-treated animals had a higher IL-4 secretion compared to the PBS-PBS-treated group. Finally, it was noted that the CXCL-1 levels were significantly higher compared to several other cytokines in the PBS-PBS-treated animals (Figure S5).

#### 3.7.2. Broncho-Alveolar Lavage (BAL) Cell Population Analysis

**Figure 7:**
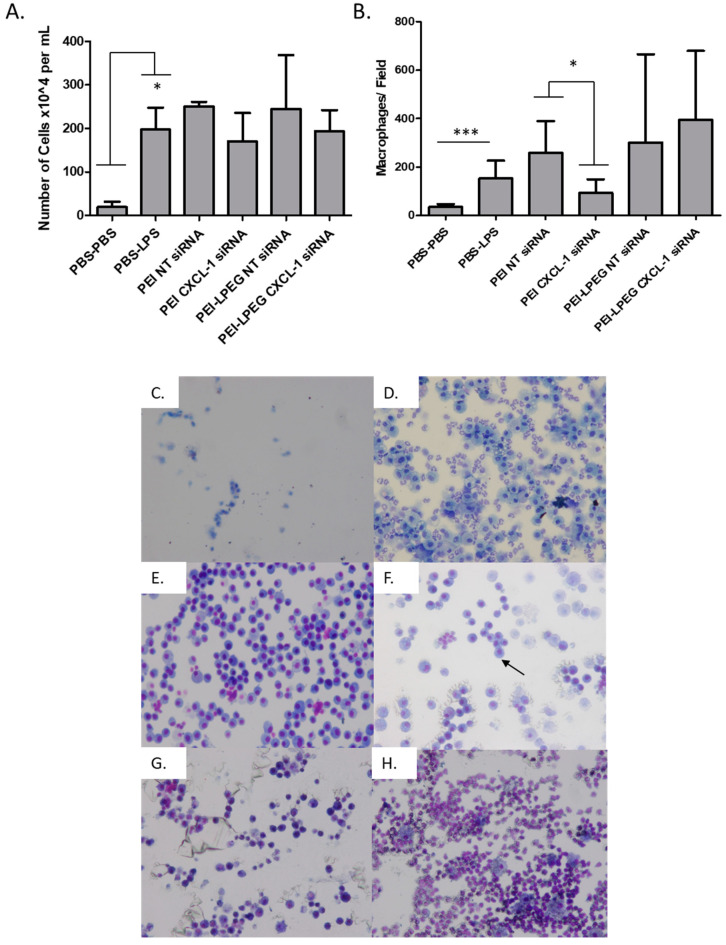
(**A**) Total cell population per mL of bronchoalveolar lavage (BAL) from the hemocytometer quantification (**B**) Macrophage population quantification from BAL image analysis (5 fields/animal) and representative images for each group including (**C**) PBS-PBS, (**D**) PBS-LPS, (**E**) PEI NT siRNA nanoparticle, (**F**) PEI CXCL-1 siRNA nanoparticle, (**G**) PEI-LPEG NT siRNA nanoparticle and (**H**) PEI-LPEG CXCL-1 siRNA nanoparticle-treated rats 24 h post-transfection. Images taken at 100× magnification (Kruskal–Wallis test and Dunn’s post-hoc test, * *p* < 0.05, *** *p* < 0.001, min of *n* = 3 ± SD).

On analysis, it was found that there was a significant increase in the overall BAL cell population following LPS stimulation, going from 19.5 (±12.26) × 10^4^ cells/mL to 197.4 (±50.82) ×10^4^ cell/mL (Figure 7A). For groups treated with the PEI and PEI-LPEG anti-IL8 siRNA nanoparticles, no significant decrease in the overall cell number was evident. A similar level of inflammation across all the LPS-treated groups was also evident upon histological analysis (Figure S6).

Using the differential cell staining of BAL samples with Eosin Y and azur/methylene blue, it was also possible to identify the separate cell populations in each treatment group (Figure 7B–H). Overall, macrophages were the predominant cell types identified in BAL. The effect of LPS stimulation on immune cell infiltration was evident, with the macrophage levels increasing from 34.8 (±12.41) macrophages per field of view in the PBS-PBS-treated group to 153.5 (±73.38) in the PBS-LPS-treated group. However, for the animals treated with the PEI anti-CXCL-1 siRNA nanoparticles, there was a significant (*p* < 0.05) decrease in macrophage infiltration compared to those treated with the PEI NT siRNA nanoparticles (93.95 ± 55.21 vs. 258.5 ± 131.9 macrophages/field, respectively). For the groups treated with PEI-LPEG siRNA nanoparticles, no significant differences in macrophage infiltration were seen between the NT and anti-CXCL-1 groups (301 ± 363 cells and 395 ± 284 cells/field, respectively) as well as when compared to the PBS-LPS-treated control group.

## 4. Discussion

The successful development of any pulmonary siRNA nanoparticle therapy is dependent on both the efficient delivery of these nanoparticles through a suitable device and the bioactivity of the delivered siRNA nanoparticles.

The effect of the vibrating mesh nebulisation on the siRNA nanoparticle size was first examined. An analysis of the effect of nebulisation on the siRNA nanoparticle size determined that the most significant changes in nanoparticle size occurred in the fractions of the TSI corresponding to the non-respirable fractions. In contrast, the nanoparticles collected in the lower (respirable) fraction of the TSI demonstrated no significant changes. While the nanoparticle size and polydispersity were high, this is most likely due to the use of PBS as a suspension buffer to facilitate nebulisation. Similarly, the use of PBS in the subsequent recovery of the particles from the TSI may have affected the size of the particles. For instance, the high ionic strength of PBS can cause electrostatically formed nanoparticles to swell considerably [23]. However, as the fractions were treated in the same manner, the relative changes in size and PDI are thus comparable. Therefore, it was encouraging to note that the PEI-LPEG particles maintained their integrity in an undiluted and physiologically relevant buffer. Furthermore, the PEI-LPEG siRNA nanoparticles demonstrated higher particle sizes than the unmodified PEI siRNA nanoparticles. This was expected since grafting the PEG molecules to PEI can result in a loss of charge density [41] and thus become more sensitive to the effects of PBS dilution. Overall, these results indicated the relatively low impact of vibrating mesh nebulisers on the integrity of the siRNA nanoparticles for pulmonary delivery.

The vibrating mesh nebuliser (VMN) performance was similarly unaffected when used for the nebulisation of the PEI/PEI-LPEG siRNA nanoparticles. The emitted droplets were all approximately 5 μm, which is in the desired size range for delivery to the lower airways [36]. However, there was a slight decrease in the output efficiency apparent when the VMN were used to nebulise the PEI-LPEG siRNA samples. This can be explained by the ability of PEG to decrease the surface tension in a solution, which can cause a reduction in the output rate. This has previously been related to the fact that samples with a low surface tension more readily wet the aperture surface and therefore decrease the efficiency of droplet formation, a phenomenon known as the “Loxy effect” [17,42].

Prior to assessing the nebulised siRNA delivery and efficacy, the IL-8 knockdown using the nanoparticles was first assessed by direct delivery onto Calu-3 monolayers to establish the sequence specificity as well as investigating any underlying inflammatory effects. When examined for changes in IL-8 protein levels, it was found that both the PEI and PEI-LPEG anti-IL8 siRNA nanoparticles were capable of eliciting decreases in IL-8 expression, but only PEI anti-IL8 siRNA significantly so and only on the basal side. Previous work with polarised epithelial cells has found that cells preferentially secrete apically [43,44]. Therefore, it is likely that apical secretion is prioritised and kept at homeostatic levels as much as possible with basal secretion bearing the decrease in IL-8 production following anti-IL-8 siRNA treatment. Furthermore, while cationic polymers can stimulate IL-8 secretion in their own right [44], the persistent presence of the PEI/PEI-LPEG free polymer in solution on the apical cell surface did not trigger any significant increases versus the PBS-treated wells. Recent studies have also highlighted that the delivery of exogenous siRNA can mediate inflammation [45,46]. However, using anti-GAPDH as a non-relevant negative control, it was also found that there were no non-specific changes in IL-8 production using either polymer.

An analysis of fluctuations in the TEER values following direct anti-IL8 siRNA nanoparticle administration revealed that treatment with both PEI and PEI-LPEG siRNA nanoparticles resulted in slight, but non-significant, decreases in TEER following transfection, with significant increases in TEER values 24 h post-transfection. This slightly greater TEER decrease seen for the PEGylated constructs has also been previously reported using PEGylated chitosan. In that study, Cassettari et al. postulated that the greater decrease in TEER values mediated by PEGylated constructs was due to the higher masses used for PEGylated polymers to reach equivalent concentrations of cationic polymer [47]. This is also in keeping with findings regarding a temporary disruption recovery in epithelial cell tight junctions following transfection [48].

Once the utility of the vibrating mesh nebuliser and sequence specificity of the anti-IL-8 siRNA nanoparticles were established, it was then possible to investigate the impact of nebulisation on the bioactivity of the siRNA nanoparticles. Previously, our lab had investigated post-nebulisation siRNA transfection using simple non-polarized Calu-3 cell cultures [23]. In this study, to better recapitulate the clinical environment, a twin-stage impinger deposition instrument was coupled with a polarised cell monolayer grown on a Transwell insert (Figure 1) for transfection studies. In the initial GAPDH validation of the system, experiments found that there were significant differences in siRNA nanoparticle behaviour going from non-nebulised to nebulised transfection. The PEI siRNA nanoparticle transfection efficiency became highly variable following nebulisation, whereas the PEI-LPEG siRNA nanoparticles were capable of consistent and significant decreases in GAPDH gene expression. While dispersing siRNA nanoparticles through a twin-stage impinger has been previously recorded [49,50], to date this has not been applied to monitor the post-nebulisation transfection efficiency.

The positive role of PEGylation in nebulisation and mucus trafficking was further demonstrated in TSI anti-IL-8 siRNA transfections of polarised Calu-3 monolayers. The PEI-LPEG siRNA-transfected cells displayed decreased IL-8 cytokine levels both apically and basally, with significant decreases in the apical secretion. In relation to the strong post-nebulisation efficiency of the PEI-LPEG samples, this is most likely as PEGylation is known to enhance colloidal stability as well as enhance the mucus penetration and trafficking of delivered particles [51,52,53]. In addition to an enhanced ability to traverse the mucus barrier, it has also been found that the aerosol administration of drugs can result in a faster trafficking time compared to solution administration. Specifically, the lower the apical fluid volume, the faster the transport [54,55]. This would explain the more obvious apical and basal IL-8 inhibition in nebulised siRNA nanoparticle-treated samples compared to the un-nebulised samples, with the mucus trafficking abilities of PEGylation becoming more quickly apparent.

Following these positive results, the siRNA nanoparticles were tested in a rat model of acute LPS-mediated inflammation. The effects of this were then examined at the genetic, protein and cellular levels. At a genetic level, the use of LPS to drive CXCL-1 up-regulation was validated with significant increases observed in gene expression. Furthermore, PEI anti-CXCL-1 siRNA nanoparticles were also found to demonstrate reduced gene expression compared to their non-targeting controls although this was not found to be significant due to the variability in the NT-siRNA-treated animals. Interestingly, it was seen that there was some disparity in the non-targeting siRNA treatment groups. The CXCL-1 gene expression in PEI NT-siRNA-LPS-treated rats was found to be strongly up-regulated, while the PEI-LPEG NT-siRNA-treated animals demonstrated CXCL-1 upregulation to a much lower degree.

Considering that the same non-targeting sequence was used in both test groups, it is unlikely that this was nucleic acid sequence dependent. The in vitro work described here using non-targeted siRNA also demonstrated that neither PEI nor PEI-LPEG elicited any significant changes in the IL-8 expression. However, it is also known that PEGylation can modulate the inflammatory effect of nanoparticles. Previous work has shown that PEI caused higher levels of complement activation, whereas conjugation with high molecular weight PEG reduced the inflammatory reactions in vivo [56,57,58]. However, it is possible that the LPS is interacting with the PEI-LPEG to some degree. Previous work has demonstrated that PEG chains with cationic moieties can neutralise LPS [59]. Combined with the increased residency time in the lungs of high molecular weight PEGylated compounds [60], this could explain why the diminished effect of LPS at a genetic level may be more pronounced in the PEI-LPEG-treated animals.

The multi-parameter cytokine screen revealed that there were few significant changes in protein expression in treated groups compared to PBS-PBS sham-treated animals. This was likely due to the very short time between LPS challenge and euthanasia of the animals (3 h). Previous work has demonstrated that it can take between 24–72 h for cytokine levels to peak in pulmonary instilled animals [39,61]. This was the case for CXCL-1 expression in all animals, with the exception of the PEI-LPEG NT siRNA-treated animals. These rats demonstrated significantly higher levels of CXCL-1 secretion when compared to the PBS-PBS-treated animals. However, the CXCL-1 levels in all the experimental groups were not significantly different from the PBS-LPS-treated animals.

However, it was also noted that there were some very high levels of expression in two out of four animals treated with PEI-LPEG siRNA. While it was not possible to say definitively what is causing this spike in CXCL-1 secretion in these animals, it is possible that animals may be reacting to the PEG moieties. There is a growing body of literature (reviewed in [62]) highlighting the risks of PEGylation in stimulating the immune system. However, the complexities of this are far from certain, with previous studies demonstrating immune responses are highly dependent on PEG chain length and grafting density. Specifically, higher levels of PEG-grafting resulted in less pro-inflammatory effects without depleting macrophages [63]. Thus, care must be taken in the future in developing low intensity dosing regimens and delivery routes such as nebulisation that minimise this outcome. Finally, intratracheally delivered PBS-PBS negative controls were also found to elicit ~40 pg/mL of CXCL-1 secretion, which is roughly equivalent to levels recorded in asthma patients (30 pg/mL) [64]. This would indicate that the procedure itself may be immune-stimulatory.

When the IFN-γ levels were examined, it was found that the administration of LPS resulted in significant increases in secretion. Interestingly, similar significant increases in IFN-γ protein expression in the PEI or PEI-LPEG-treated groups were not observed following LPS administration, regardless of the siRNA type. This was in line with studies which have found that PEI has a lower stimulatory effect on IFN-γ expression than other polymers in mice [39,65].

In addition to cytokine content, the cellular composition in the extracted BAL fluid was investigated. Here, it was found that the administration of LPS resulted in a rapid and significant influx of a variety of immune cells. From the total cell population counts of the extracted BAL fluid, there were no significant differences between all the LPS-treated sample groups. However, differential staining for macrophages revealed that the delivery of anti-CXCL-1 siRNA could elicit a significant reduction using PEI nanoparticles. This further validates the in vitro evidence from this study demonstrating the higher rate of transfection with PEI when directly administered to cells. This may also be aided by the fact that PEI has also been documented as depleting BAL macrophages when administered to the lungs [63] (although no evidence of this was observed in NT-siRNA-treated rats).

Given the large influx of innate immune cells, an important consideration for future siRNA-based inflammatory therapies in the lungs would be to enable targeting of both the epithelial cells and macrophages. This would allow for a more complete inhibition of the inflammatory response. This is currently being investigated in our own lab and elsewhere [66,67,68] and must be partnered with advanced 3D cell culture models for enhanced in vitro assessment [69].

Considering the in vitro/in vivo disparity in efficacy of PEI-LPEG nanoparticles in significantly decreasing IL-8/CXCL-1 at the genetic level and indications of high levels of cytokine secretion in vivo, it is important to examine the differences between the nebulised and intratracheal delivery of siRNA nanoparticles.

Specifically, in terms of invasiveness and direct delivery, intratracheal delivery was deemed necessary due to the high levels of siRNA that would be required for in vivo nebulisation (a previous study of nebulised mRNA at 0.5 mg/mL [21]) combined with the technical challenges associated with nebulised delivery to small animals. As a result of this, the N/P ratios that were optimised for nebulisation and had demonstrated efficient knockdown were now delivered directly to the lungs in a much more concentrated fashion. This concentrated dose would further exacerbate any underlying inflammation associated with siRNA nanoparticle delivery. This is in agreement with a recent study by Ng et al. which reported no differences between the bolus and intra-tracheal delivery of siRNA in mice [45]. Therefore, the benefits of PEGylation observed in the study were very much dependent on their means of delivery and may have been obscured by intra-tracheal/bolus administration in vivo.

There is growing evidence now demonstrating delivery-related inflammatory responses to the bolus delivery of siRNA [56,70] (indications of which were present in BAL fluid). Complement Activation Related Pseudo-allergy (CARPA) has triggered a move towards lower density/more controlled delivery methods such as i.v. perfusion for continued clinical development [5]. Similarly, this study has demonstrated that nebulisation represents an analogous low-density, aerosol approach to achieving high levels of transfection efficiency while avoiding inflammatory events associated with bolus delivery. Future work for pulmonary delivery of siRNA (and all oligonucleotide therapeutics) must now focus on not reverting to bolus delivery methods when progressing to pre-clinical studies. The continued development and refinement of existing pre-clinical models of nebulisation is a priority in this case.

## 5. Conclusions

In this study, convergent device-nanoparticle development was addressed through the in vitro testing of knockdown efficiency of nebulised siRNA nanoparticles in successively more complex cell assays. In parallel, the effect of siRNA nanoparticle cargo on the nebuliser output and performance was also assessed and vice versa. Finally, these siRNA nanoparticles were progressed to in vivo testing in an LPS-induced rat model of acute pulmonary inflammation.

Through these experiments, it was determined that siRNA nanoparticles are capable of being efficiently nebulised through a vibrating mesh nebuliser without any impact on the integrity of the nanoparticles or the device performance. Twin-stage impinger cell studies highlighted the significantly enhanced ability of PEI-PEG siRNA nanoparticles to be nebulised, traverse the mucus lining of airway epithelial cells and reduce gene and protein expression compared to PEI siRNA nanoparticles.

To support in vivo testing, a rat model of acute pulmonary inflammation was established. The CXCL-1 gene expression could be reduced using some of the siRNA nanoparticle treatments to a level comparable to negative (healthy) controls. Furthermore, in the case of PEI anti-CXCL-1 siRNA nanoparticle-treated animals, significant reductions in macrophage infiltration were also observed. However, there was significant variability and inconsistency in the effects seen for the treatment groups on CXCL-1 protein expression. In this study we have noted initial evidence of a relationship between the efficacy of the PEGylated PEI and the means of delivery. Specifically, issues were identified when switching to direct intubation and bolus delivery instead of nebulisation, as was used in the in vitro studies. The genetic knockdown elicited by some of the treatments may well be realised at a protein level with further refinement in dosing, dose timing and the application of a less invasive means of delivery in future studies.

## Figures and Tables

**Table 1:** Effect of PEI and PEI-LPEG siRNA nanoparticles on the vibrating mesh nebuliser output rate (mL/min), % fine particle fraction (%FPF) and by volumetric median diameter (Dv (50)).

Particle	Surface Tension (dyn/cm)	Output (mL/min)	%FPF	Dv (50)
PBS	62.03 ± 0.56	0.417	58.25	5.25
PEI-siRNA N/P = 15	68.57 ± 0.15	0.409	56.47	5.31
PEI-LPEG-siRNA N/P = 15	59.87 ± 0.20	0.281	57.56	5.36

## Supplementary Materials

The following are available online at https://www.mdpi.com/2079-4991/10/7/1248/s1, Figure S1: GPC size and purity analysis of synthesized PEI-LPEG overlaid with its respective starting materials., Figure S2: PEI-LPEG carboxylic bonding presence was shown in ^13^C NMR at 164 ppm, Figure S3: ^1^H NMR of synthesized PEI-LPEG polymer with PEI present at 2.5 ppm and PEG present at 3.5 ppm, Figure S4: Rat Demonstration 7-Plex Ultra-Sensitive Kit analysis of inflammatory cytokine responses elicited by intratracheal instillation of PBS-PBS, PBS-LPS, non-targeting (NT) or anti-IL-8 siRNA nanoparticles in a rat model. (A) interferon-γ (IFN-γ) (B) interleukin-1β (IL-1β) (C) IL-13 (D) IL-4 (E) IL-5 and (F) Tumor necrosis factor alpha (TNF-α) (significance vs. PBS-LPS treated samples, Kruskal-Wallis test and Dunn’s post-hoc test, min of n = 3 ± SD, ** *p* < 0.01), Figure S5: BAL cytokine expression levels in PBS-PBS treated rats (minimum n = 3 ±SD, Kruskal-Wallis test and Dunn’s post-hoc test, * *p* < 0.05), Figure S6: Pulmonary histopathology semi quantitative scoring of neutrophil-rich inflammation. Haemotoxylin and eosin stained lung sections from top PBS, PBS-LPS and siRNA nanoparticle treated rats were scored based on the degree of neutrophil-rich inflammation observed (− absent, + mild, ++ moderate and +++ highly inflamed). Images were acquired at 40×, 200× and 600× magnification with arrows indicating evidence of inflammation and of blood and protein in the alveoli and loss of the alveolar lining at higher levels of severity.
