# Supplementary material for: In Vitro and In Vivo Assessment of PEGylated PEI for Anti-IL-8/CxCL-1 siRNA Delivery to the Lungs
Source: Nanomaterials (Basel). 2020 Jun 27;10(7):1248. doi: 10.3390/nano10071248 (PMC7407419; doi:10.3390/nano10071248)
Supplement: Supplementary file 1 [file nanomaterials-10-01248-s001.pdf]

nanomaterials

Article

In Vitro and In Vivo Assessment of PEGylated PEI for Anti-IL-8/CxCL-1 siRNA Delivery to the Lungs

Alan J Hibbitts [1,2], Joanne M Ramsey [1], James Barlow [3], Ronan MacLoughlin [1,4,5] and Sally-Ann Cryan [1,2,*]

1 School of Pharmacy & Biomolecular Sciences, Royal College of Surgeons in Ireland, Dublin, D02 YN77, Ireland; alanhibbitts@rcsi.ie (A.J.H.); ramseyj@tcd.ie (J.M.R.); RMacLoughlin@aerogen.com (R.M.)
2 Trinity Centre for Biomedical Engineering, Trinity College, Dublin, D02 R590, Ireland
3 Dept of Chemistry, Royal College of Surgeons in Ireland, Dublin, D02 YN77, Ireland; jambarlow@rcsi.ie
4 School of Pharmacy and Pharmaceutical Sciences, Trinity College, Dublin, D02PN40, Ireland
5 Aerogen Ltd. Galway Business Park, Galway, H91 HE94, Ireland
* Correspondence: scryan@rcsi.ie; Tel.: +353-14022741

Supplementary Materials:

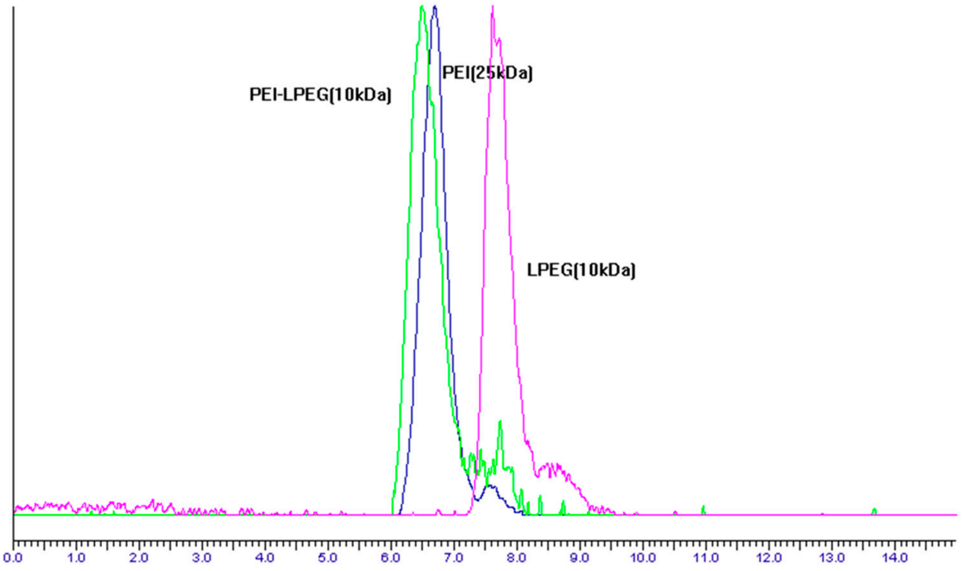

Figure S1. GPC size and purity analysis of synthesised PEI-LPEG overlaid with its respective starting materials.

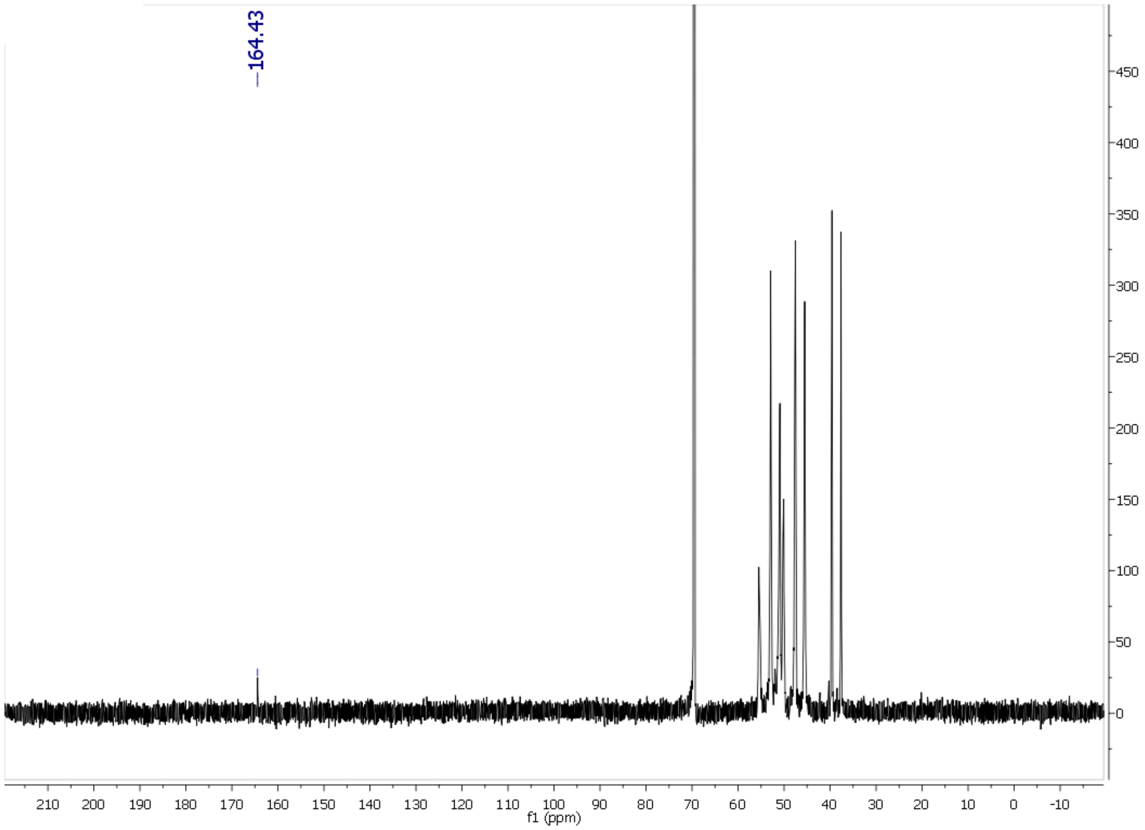

Figure S2. PEI-LPEG carboxylic bonding presence was shown in ^{13}C NMR at 164 ppm.

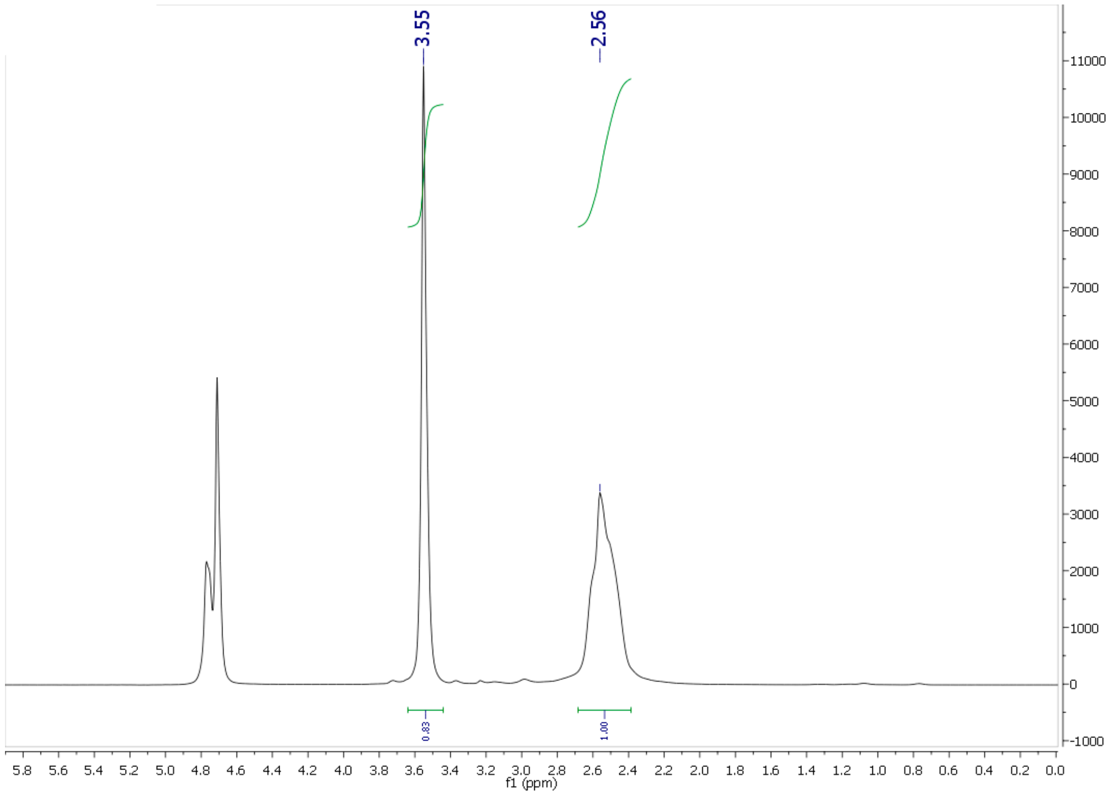

Figure S3. ^{1}H NMR of synthesised PEI-LPEG polymer with PEI present at 2.5 ppm and PEG present at 3.5 ppm.

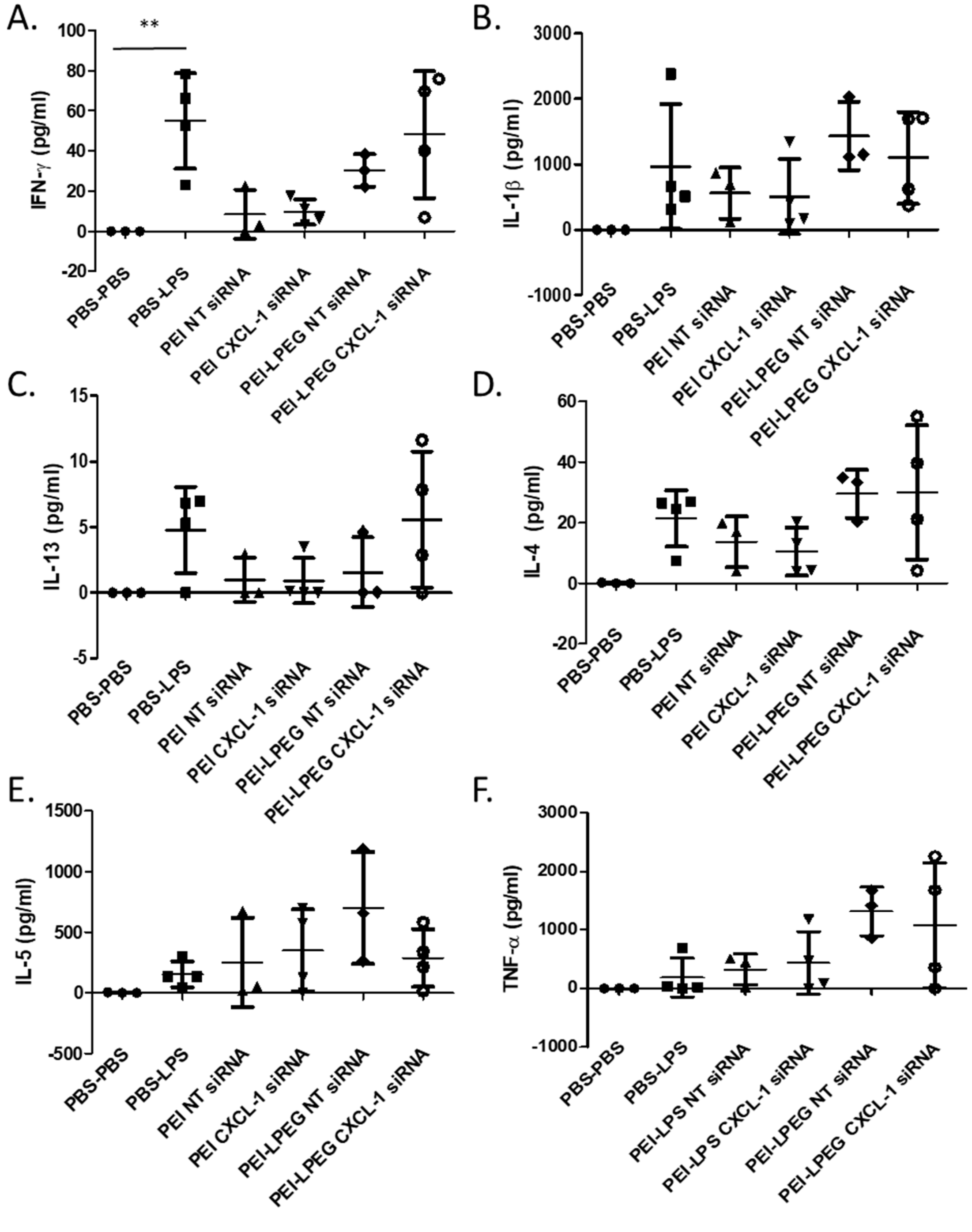

Figure S4. Rat Demonstration 7-Plex Ultra-Sensitive Kit analysis of inflammatory cytokine responses elicited by intratracheal instillation of PBS-PBS, PBS-LPS, non-targeting (NT) or anti-CXCL-1 siRNA nanoparticles in a rat model. (**A**) interferon-γ (IFN- γ) (**B**) interleukin-1β (IL-1β) (**C**) IL-13 (**D**) IL-4 (**E**) IL-5, and (**F**) umour necrosis factor alpha (TNF-α) (significance vs. PBS-LPS treated samples, Kruskal-Wallis test and Dunn's post-hoc test, min of $n = 3 \pm$ SD, ** $p < 0.01$).

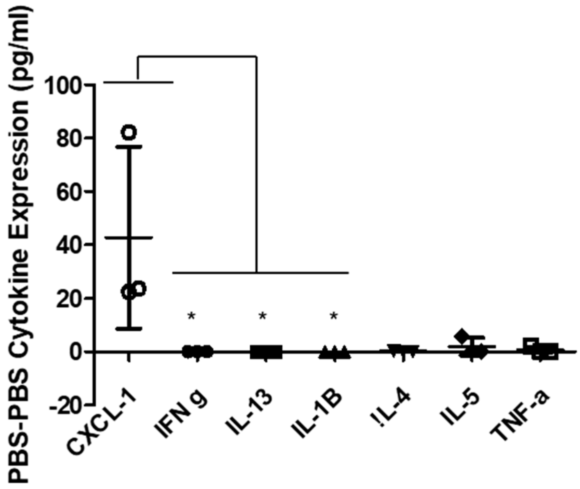

Figure S5. BAL cytokine expression levels in PBS-PBS treated rats (minimum $n = 3 \pm SD$, Kruskal-Wallis test and Dunn's post-hoc test, * $p < 0.05$).

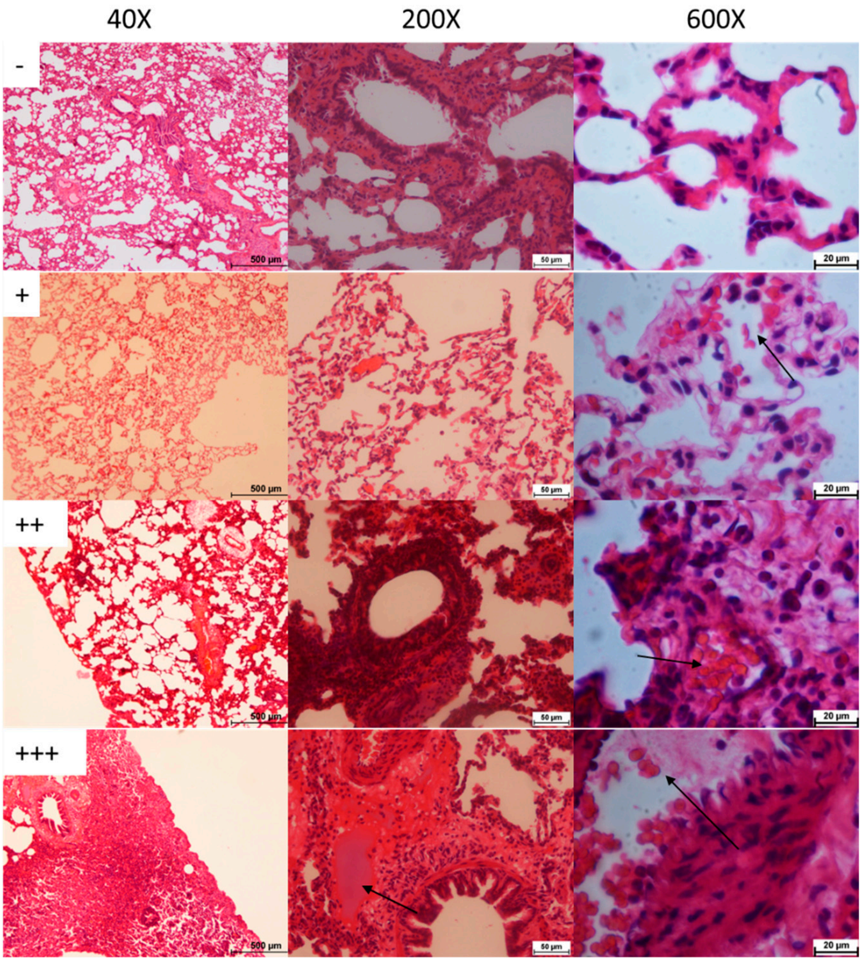

Figure S6. Pulmonary histopathology semi quantitative scoring of neutrophil-rich inflammation. Haemotoxylin and eosin stained lung sections from top PBS, PBS-LPS and siRNA nanoparticle treated rats were scored based on the degree of neutrophil-rich inflammation observed (− absent, + mild, ++ moderate and +++ highly inflamed). Images were acquired at 40×, 200× and 600× magnification with

arrows indicating evidence of inflammation and of blood and protein in the alveoli and loss of the alveolar lining at higher levels of severity.

© 2020 by the authors. Submitted for possible open access publication under the terms and conditions of the Creative Commons Attribution (CC BY) license (http://creativecommons.org/licenses/by/4.0/).
